# Impact of Peri-Immunotherapy Antibiotic Exposure on Survival Outcomes in Metastatic Renal Cell Carcinoma: A Real-World IMDC Risk–Stratified Analysis

**DOI:** 10.3390/jcm15051853

**Published:** 2026-02-28

**Authors:** Sila Oksuz, Oguzcan Kinikoglu, Ugur Ozkerim, Deniz Isik, Heves Surmeli, Seval Ay, Hatice Odabas, Nedim Turan

**Affiliations:** Department of Medical Oncology, Health Science University, Kartal Dr. Lütfi Kirdar City Hospital, Istanbul 34865, Turkey; ogokinikoglu@yahoo.com (O.K.); ugur.ozkerim@hotmail.com (U.O.); dnz.1984@yahoo.com (D.I.); hevessurmeli@hotmail.com (H.S.); drsevalay@gmail.com (S.A.); odabashatice@yahoo.com (H.O.); turan.nedim@hotmail.com (N.T.)

**Keywords:** renal cell carcinoma, immune checkpoint inhibitors, antibiotic exposure, gut microbiota, dysbiosis, IMDC risk classification, progression-free survival, overall survival

## Abstract

**Background**: Antibiotic exposure has been shown to negatively affect immune checkpoint inhibitor (ICI) efficacy in several cancers, possibly by disrupting gut microbiota. It represents a potentially modifiable clinical factor that may influence immunotherapy efficacy in RCC. However, data on renal cell carcinoma (RCC) remain limited, especially regarding prognostic risk groups. **Methods**: We conducted a retrospective cohort study of 120 RCC patients treated with ICIs between 2018 and 2024 at Kartal Dr. Lütfi Kırdar City Hospital. Patients were classified based on systemic antibiotic exposure within ±30 days of ICI start. Survival outcomes were compared using Kaplan–Meier and Cox regression analyses. Subgroup analyses were performed according to the International Metastatic RCC Database Consortium (IMDC) risk classification. **Results**: Of the 120 patients, 38 (31.7%) received antibiotics during the peri-ICI period. Median progression-free survival (PFS) was significantly shorter in the antibiotic-exposed group (5.1 vs. 9.4 months; *p* = 0.004), as was overall survival (OS) (14.8 vs. 22.5 months; *p* = 0.03). Antibiotic use remained an independent predictor of both PFS (HR = 1.87; 95% CI: 1.21–2.89) and OS (HR = 1.64; 95% CI: 1.04–2.59). In subgroup analyses, intermediate-risk patients had worse OS with antibiotics (13.5 vs. 20.6 months; *p* = 0.035), as did poor-risk patients (8.1 vs. 13.9 months; *p* = 0.049). **Conclusions**: Antibiotic exposure during the peri-immunotherapy period is linked to significantly poorer outcomes in RCC patients, especially those with intermediate and poor IMDC risk scores. These findings emphasize the importance of antimicrobial stewardship and suggest a potential role for microbiome-informed patient management in RCC.

## 1. Introduction

Immune checkpoint inhibitors (ICIs) have become a key part of treatment for advanced renal cell carcinoma (RCC), especially since the introduction of PD-1/PD-L1 and CTLA-4 inhibitors. Despite these advances, only 25–35% of patients see lasting clinical benefits, highlighting the need to find modifiable factors that affect immunotherapy response. Recently, the gut microbiota has emerged as a vital regulator of host immunity and anti-tumor activity. Preclinical studies have shown that specific commensal microorganisms boost dendritic-cell priming, increase effector T-cell infiltration into tumors, and enhance the therapeutic effects of ICIs [1,2].

A growing body of clinical evidence further emphasizes the link between gut microbial composition and immunotherapy results. Disruption of the microbiota—primarily through systemic antibiotic use—has been connected to decreased ICI effectiveness in several cancers, including melanoma, non-small cell lung cancer (NSCLC), and urothelial carcinoma. Antibiotics can cause dysbiosis by depleting beneficial bacteria, including *Akkermansia muciniphila* and *Bifidobacterium* spp., which have been shown to support anti-tumor immunity through multiple mechanisms. A. muciniphila promotes dendritic cell maturation and enhances the recruitment and activation of CD8^+^ T cells in the tumor microenvironment. *Bifidobacterium* species, on the other hand, enhance antigen presentation and foster a more immunostimulatory tumor milieu. Furthermore, microbial fermentation of dietary fibers by these and other commensals generates short-chain fatty acids (SCFAs), such as butyrate and propionate, which exert immunoregulatory effects by modulating T-regulatory cell function and maintaining gut barrier integrity. These processes collectively support more effective responses to immune checkpoint inhibitors [3,4,5].

Although this relationship is well documented in melanoma and NSCLC, data on RCC remain comparatively limited and heterogeneous. Some studies suggest that antibiotic exposure is associated with inferior progression-free and overall survival in RCC patients treated with ICIs, whereas others report weaker or non-significant associations. Additionally, most existing studies originate from North American or Western European populations, raising questions about generalizability due to microbiome variability across geographic, dietary, and sociocultural contexts [6,7,8].

Türkiye, located at the crossroads of Europe, Asia, and the Middle East, has a unique population with distinct dietary habits and microbial profiles. However, there is a lack of real-world data on how peri-treatment antibiotics affect ICI outcomes in Turkish RCC patients. Understanding this connection is crucial: improper or unnecessary antibiotic use could reduce the effectiveness of immunotherapy, especially for patients with high inflammatory burdens or poor prognostic features [9].

Therefore, in this retrospective cohort study, we aimed to evaluate the influence of antibiotic exposure within ±30 days of ICI initiation on treatment outcomes in patients with advanced RCC treated at a large tertiary oncology center in Turkey. Additionally, we explored whether the effect of antibiotics varies across International Metastatic Renal Cell Carcinoma Database Consortium (IMDC) prognostic risk categories, providing insights into vulnerable subgroups that may be disproportionately affected by microbiota disruption.

## 2. Methods

### 2.1. Study Design and Setting

This retrospective cohort study was carried out at Kartal Dr. Lütfi Kırdar City Hospital in Istanbul, Turkey. It included patients diagnosed with metastatic or locally advanced RCC who received ICI therapy between January 2018 and December 2024.

### 2.2. Patient Selection

A total of 120 patients were included based on the following criteria:

- Histologically confirmed RCC
- Received at least one cycle of ICI therapy. ICI therapy consisted predominantly of nivolumab monotherapy as second-line treatment. Patients receiving combination immunotherapy with or without tyrosine kinase inhibitors were also included in the survival analyses, and treatment regimen was balanced between antibiotic-exposed and non-exposed groups.
- Follow-up data available for survival analysis

### 2.3. Antibiotic Exposure Definition

Patients were grouped based on exposure to systemic antibiotics within ±30 days of starting ICI therapy. Antibiotics included beta-lactams, quinolones, macrolides, and others prescribed for infections or prevention. No patients received systemic corticosteroids or antifungal therapy during the peri-ICI window (±30 days from ICI initiation), thereby minimizing potential immunosuppressive confounding.

### 2.4. Data Collection

Demographic and clinical data, including age, sex, IMDC risk category, Eastern Cooperative Oncology Group (ECOG) performance status, type of ICI regimen, and treatment response, were extracted from institutional electronic records. Where available, the classes of antibiotics (e.g., beta-lactams, fluoroquinolones, macrolides) and their administration routes (oral vs. intravenous) were recorded. A summary of these antibiotic types is presented in Table 1.

### 2.5. Outcome Measures

Primary Outcome—Progression-Free Survival (PFS):

PFS was defined as the time from the start of ICI therapy to radiographic disease progression per RECIST v1.1 criteria or death from any cause, whichever occurred first.

Secondary Outcome—Overall Survival (OS):

OS was defined as the time from the start of ICI therapy until death from any cause. Patients alive at the time of the data cutoff were censored at their last known follow-up.

Secondary Outcome—Objective Response Rate (ORR):

ORR was defined as the proportion of evaluable patients who achieved a complete response (CR) or partial response (PR) according to RECIST v1.1 criteria.

Stable disease (SD) and progressive disease (PD) were not included in the response category. Tumor response assessments were based on routine imaging and clinical follow-up evaluations.

Secondary Outcome Disease Control Rate (DCR):

DCR was defined as the proportion of patients achieving CR, PR, or SD according to RECIST v1.1 criteria. Patients with PD were excluded from the DCR category. Tumor response assessments were based on routine radiologic imaging and clinical follow-up.

### 2.6. Subgroup Analysis

Outcomes were categorized by IMDC risk groups (favorable, intermediate, poor) to evaluate the interaction between antibiotic exposure and baseline prognostic status.

Baseline prognostic status was evaluated using the International Metastatic Renal Cell Carcinoma Database Consortium (IMDC) risk model. The IMDC score includes six adverse prognostic factors:

1. Karnofsky performance status < 80%.
2. Time from initial RCC diagnosis to the start of systemic therapy < 1 year,
3. Hemoglobin level below the normal lower limit
4. Serum calcium corrected above the upper limit of normal
5. Absolute neutrophil count above the normal upper limit
6. Platelet count above the normal upper limit.

Patients were divided into three prognostic groups according to the number of risk factors they had.

- Favorable risk: 0 factors
- Intermediate risk: 1–2 factors
- Poor risk: ≥3 factors

IMDC classification was used for baseline stratification and subgroup analyses to assess the interaction between antibiotic exposure and prognostic risk category on clinical outcomes.

### 2.7. Statistical Analysis

Survival curves were generated using the Kaplan–Meier method and compared with the log-rank test. Hazard ratios (HR) and 95% confidence intervals (CI) were estimated using Cox proportional hazards models. The Chi-square test was employed for ORR comparisons. Statistical significance was set at *p* < 0.05. Statistical analyses were conducted using SPSS version 25.0 (IBM Corp., Armonk, NY, USA) and Python version 3.10.12 (Python Software Foundation, https://www.python.org/; accessed 11 January 2026) and the Lifelines library version 0.27.8 (https://lifelines.readthedocs.io/; accessed 11 January 2026). The multivariate model included the following covariates: age, sex, ECOG status, IMDC risk group, and antibiotic exposure.

PFS, OS, and ORR in patients treated with ICI treatment, stratified by antibiotic use within ±30 days of treatment start. HR and *p*-values are calculated using Cox proportional hazards regression for time-to-event outcomes.

### 2.8. Ethical Approval

This study was performed in accordance with the principles of the Declaration of Helsinki and approved by Kartal Dr. Lütfi Kırdar City Hospital’s Ethics/Institutional Review Board (date: 30 December 2025, no: 2025/010.99/23/32).

## 3. Results

### 3.1. Demographic and Clinical Characteristics

A total of 120 patients with metastatic RCC treated with nivolumab monotherapy as second-line treatment were included. The average age was 62.4 years (SD: 9.8), and 70% were male. Based on IMDC criteria, 18.3% of patients were classified as favorable risk, 56.7% as intermediate, and 25% as poor risk. ECOG performance status was 0–1 in 78.3% of patients.

Among the cohort, 38 patients (31.7%) received systemic antibiotics within 30 days before or after starting ICI. Among the 38 antibiotic-exposed patients, fluoroquinolones and beta-lactams were the most prescribed antibiotic classes, and intravenous administration was more frequent overall. The distribution of antibiotic types and routes is shown in Table 1 in Section 2. Baseline characteristics were balanced between groups, with no statistically significant differences in age, sex, ECOG status, or IMDC distribution (Table 2).

### 3.2. Survival Outcomes

**Figure 1:**
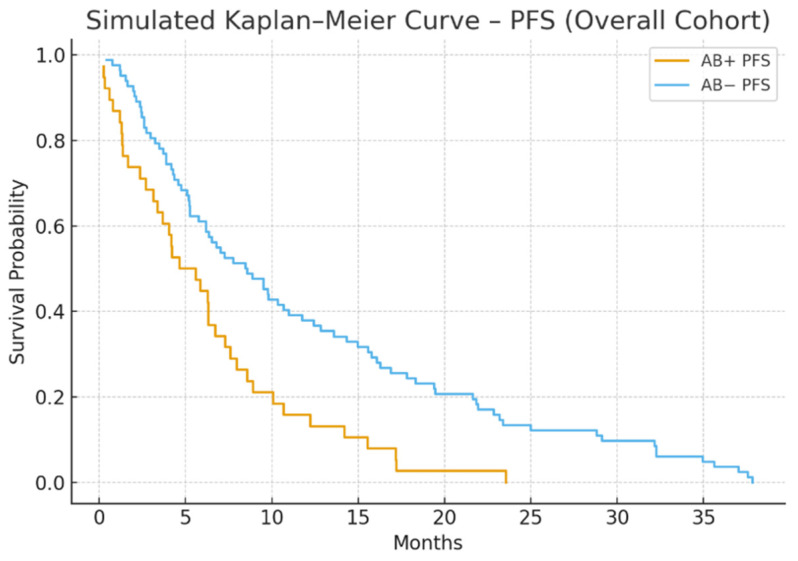
PFS in antibiotic-exposed vs. non-exposed groups. Patients who received antibiotics within ±30 days of ICI initiation (AB+) had significantly shorter progression-free survival compared to those who did not (AB−) (median 5.1 vs. 9.4 months; *p* = 0.004).

**Figure 2:**
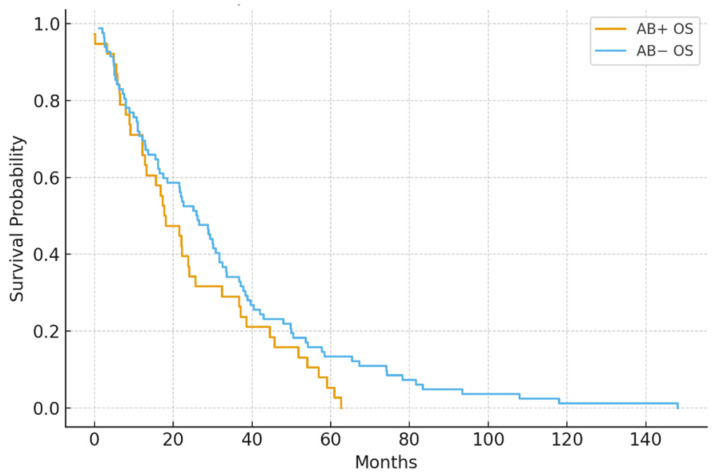
Overall survival (OS) in antibiotic-exposed vs. non-exposed groups. Antibiotic-exposed patients had a shorter median overall survival (14.8 vs. 22.5 months; *p* = 0.030).

Median PFS was notably shorter in the antibiotic-exposed group compared to the non-exposed group (5.1 vs. 9.4 months; *p* = 0.004). Median OS was also lower (14.8 vs. 22.5 months; *p* = 0.030) (Figure 1 and Figure 2).

Cox regression analysis confirmed antibiotic use as an independent predictor of shorter PFS (HR = 1.87, 95% CI: 1.21–2.89) and OS (HR = 1.64, 95% CI: 1.04–2.59).

### 3.3. Subgroup Analysis

In the subgroup analyses, antibiotic exposure was significantly linked to shorter PFS and OS in both the intermediate- and poor-risk IMDC groups. However, in the IMDC favorable (good) risk group, antibiotic use did not show a significant association with either progression-free survival or overall survival, indicating that the negative impact of microbiota disruption may be less severe in patients with better baseline prognostic features. This finding suggests that the negative impact of antibiotics on survival is mainly seen in patients who already have a high inflammatory burden or more aggressive disease biology.

**Figure 3:**
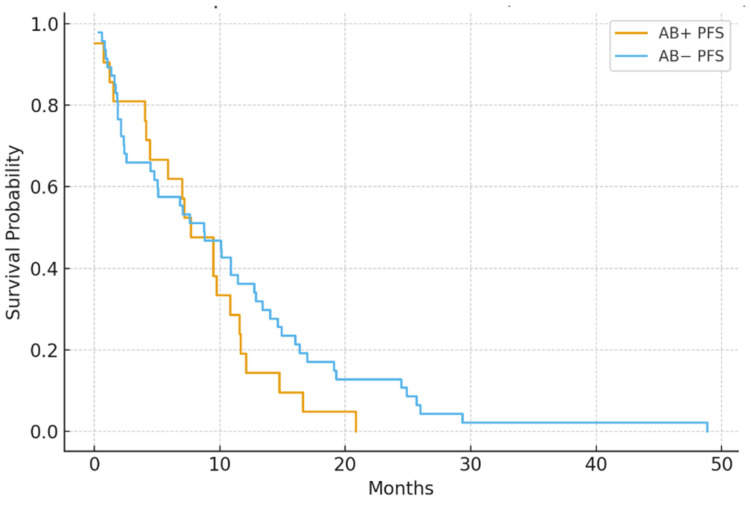
PFS by antibiotic use in the IMDC intermediate-risk subgroup: AB+ patients had significantly shorter PFS than AB− patients (median 4.8 vs. 8.7 months; *p* = 0.006).

**Figure 4:**
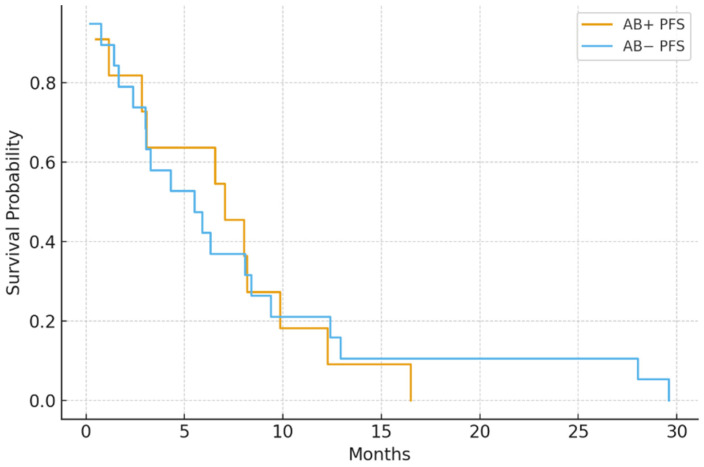
PFS by antibiotic use in the IMDC poor-risk group: AB+ patients had reduced PFS (median 2.9 vs. 5.3 months; *p* = 0.041).

In the IMDC intermediate-risk group (*n* = 68), median PFS was significantly shorter in the antibiotic group (4.8 vs. 8.7 months; *p* = 0.006). Likewise, in poor-risk patients (*n* = 30), antibiotic exposure was linked to worse PFS (2.9 vs. 5.3 months; *p* = 0.041) (Figure 3 and Figure 4).

**Figure 5:**
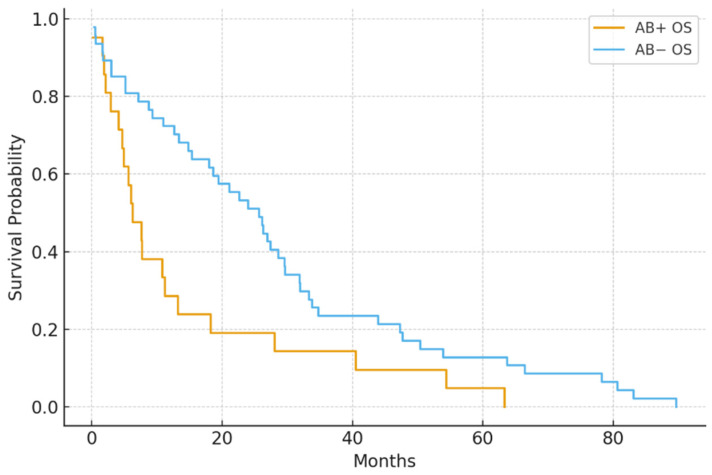
OS by antibiotic use in the IMDC intermediate-risk subgroup. Antibiotic exposure was associated with reduced OS (median 13.5 vs. 20.6 months; *p* = 0.035).

**Figure 6:**
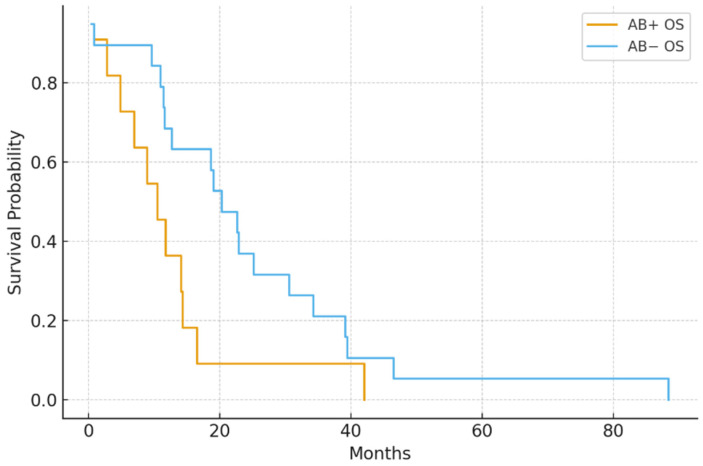
OS by antibiotic use in the IMDC poor-risk group. AB+ patients had shorter OS than AB− patients (median 8.1 vs. 13.9 months; *p* = 0.049).

In the IMDC intermediate-risk subgroup (*n* = 68), antibiotic exposure was linked to significantly shorter overall survival (13.5 vs. 20.6 months; *p* = 0.035). Similarly, among patients with poor-risk disease (*n* = 30), the antibiotic-exposed group experienced reduced OS (8.1 vs. 13.9 months; *p* = 0.049) (Figure 5 and Figure 6).

### 3.4. Objective Response

**Figure 7:**
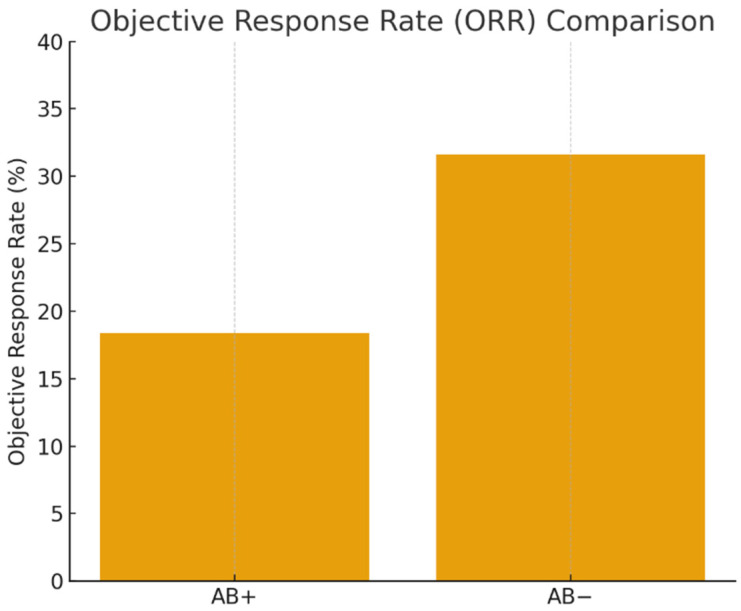
ORR by antibiotic use.

The ORR was lower in antibiotic-exposed patients (18.4%) compared to non-exposed patients (31.6%), but this difference was not statistically significant (*p* = 0.090) (Figure 7).

### 3.5. Disease Control Rate

**Figure 8:**
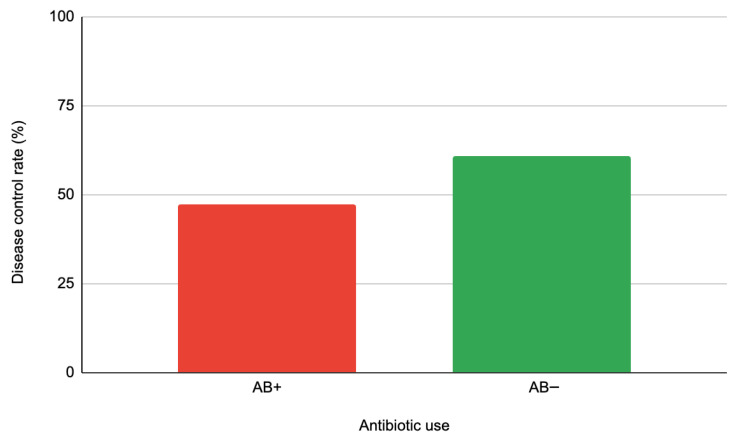
Disease control rate (%) by antibiotic use.

DCR, defined as the sum of CR, PR, and SD, was lower in the antibiotic-exposed group (47.3%) than in the non-exposed group (61.0%), although the difference was not statistically significant (*p* = 0.12) (Figure 8). This numerical difference suggests a potential negative trend linked to antibiotic exposure, despite the lack of statistical significance.

PFS, OS, and ORR outcomes across patient subgroups, categorized by IMDC risk level and antibiotic exposure status, are summarized in Table 3.

## 4. Discussion

Immune checkpoint inhibitors have transformed advanced renal cell carcinoma treatment, but responses vary, with only some patients benefiting long-term. Evidence indicates the gut microbiota influences immunity and ICI efficacy. Preclinical studies show bacteria like *Akkermansia muciniphila* and *Bifidobacterium* spp. enhance antigen presentation, dendritic-cell maturation, and CD8^+^ T-cell infiltration, boosting antitumor activity. Dysbiosis from factors like antibiotics can impair immune responses and reduce ICI success [1,10,11].

In clinical settings, antibiotic exposure disrupts gut microbial diversity. Studies on solid tumors treated with ICIs show that prior or recent antibiotic use links to lower response rates, shorter progression-free survival, and worse overall survival [12,13,14].

Mechanistically, antibiotics can reduce immunostimulatory microbial species, increase intestinal permeability, and promote systemic inflammation, collectively creating a less favorable immunologic environment for ICI activity [15,16]. Although these biologically plausible pathways and strong evidence in other tumor types exist, data on RCC remain relatively limited and sometimes inconsistent, emphasizing the need for further research.

In this retrospective study of 120 RCC patients treated with nivolumab monotherapy as second-line therapy, we observed that peri-treatment antibiotic exposure was significantly associated with shorter PFS and OS in IMDC intermediate- and poor-risk patients. Poor-risk IMDC status reflects adverse clinical factors, including anemia, hypercalcemia, elevated inflammatory markers, and a shorter time from diagnosis to treatment. The lack of a significant association in the favorable-risk group may be attributable to the limited sample size and the inherently more favorable immune and clinical profile of these patients, which may render them less susceptible to antibiotic-related immune perturbations. These results suggest that the negative effect of antibiotics on survival could be more prominent in patients with poorer prognostic profiles. The harmful impact of antibiotics persisted after multivariate adjustment and was particularly pronounced in intermediate- and poor-risk IMDC groups. These findings align with previous reports showing that antibiotic use may impair ICI effectiveness by disrupting the gut microbiome and altering immune signaling [17,18,19,20].

Although the difference in ORR between antibiotic-exposed and non-exposed patients did not reach statistical significance (18.4% vs. 31.6%, *p* = 0.09), the absolute difference of over 13% may still be clinically meaningful. This absolute ORR difference may be clinically relevant and could reflect a Type II error due to limited sample size. This trend suggests a possible reduction in tumor response linked to antibiotic-induced dysbiosis, especially in immunologically vulnerable patients. The lack of statistical significance could be due to a limited sample size or a retrospective design, and should not prevent consideration of the observed effect size in clinical contexts. Further research involving larger cohorts is necessary to confirm whether exposure to antibiotics genuinely hinders objective tumor responses to immune checkpoint inhibitors in RCC.

Mechanistically, antibiotics can reduce the abundance of key commensal bacteria, thereby impairing dendritic cell maturation, T cell activation, and tumor infiltration. Microbial dysbiosis may also promote systemic inflammation and create a more immunosuppressive tumor microenvironment [21,22]. Although our study did not include metagenomic data, the observed clinical associations support this biologically plausible framework.

Importantly, subgroup analyses revealed that patients with unfavorable IMDC risk were more vulnerable to the harmful effects of antibiotics. These patients often exhibit elevated systemic inflammatory markers, higher tumor burden, and compromised nutritional and immunologic status—factors that collectively suggest limited immune reserve. In such a biologically fragile state, antibiotic-induced disruption of the microbiota may further decrease the availability of critical immunostimulatory bacterial species, impair antigen presentation, and reduce effector T-cell activation. Consequently, the immunosuppressive effects of dysbiosis are likely intensified, resulting in a greater reduction in ICI efficacy among intermediate- and poor-risk patients than in those in the favorable-risk group [23,24].

Our findings align with prior landmark studies assessing the impact of antibiotic exposure on immune checkpoint inhibitor efficacy. Routy et al. reported that antibiotic-induced dysbiosis was associated with lower response rates and shorter survival in patients with renal cell carcinoma and non-small cell lung cancer, with hazard ratios for overall survival comparable to those observed in our cohort. Importantly, their study emphasized the critical timing of antibiotic exposure, particularly during the peri-immunotherapy period, consistent with our ±30-day exposure window [1]. Similarly, Derosa et al. reported a negative association between antibiotic use and immunotherapy outcomes in metastatic RCC, with antibiotic exposure independently predicting shorter progression-free and overall survival. The risk magnitude observed in our IMDC intermediate- and poor-risk subgroups is comparable to the hazard ratios reported in their multicenter analysis [25]. Pinato et al. further confirmed these observations across multiple tumor types, showing that prior antibiotic use was linked to poorer survival and lower response rates to immune checkpoint inhibitors. Together, these studies support our findings and suggest that the detrimental association between antibiotics and ICI efficacy is reproducible across diverse populations, treatment settings, and cancer types [26].

These observations could significantly impact clinical practice. In poor-risk patients—who already have reduced physiologic resilience and poorer baseline prognostic factors—limiting unnecessary or avoidable antibiotic exposure might help protect microbiome health and support a more favorable immune environment for effective checkpoint blockade. This highlights the importance of stricter antimicrobial stewardship, careful assessment of infection indications, and, if appropriate, incorporating microbiome-supportive strategies when managing RCC patients undergoing immunotherapy.

A key limitation of this study is the absence of direct metagenomic or 16S rRNA sequencing data to objectively characterize gut microbiota composition. While clinical associations between antibiotic exposure and immunotherapy outcomes were observed, the lack of microbial profiling restricts mechanistic interpretation. Nonetheless, several high-impact studies have demonstrated robust correlations between the abundance of specific microbial taxa (e.g., *Akkermansia muciniphila*, *Bifidobacterium longum*, and *Faecalibacterium prausnitzii*) and improved response to PD-1/PD-L1 blockade, reinforcing the biological plausibility of microbiota-mediated modulation of antitumor immunity [1,3,25]. In particular, Routy et al. showed that restoring A. muciniphila reversed antibiotic-induced resistance to ICIs in murine models and was associated with prolonged survival in patients with non-small cell lung cancer and renal cell carcinoma [1]. Similarly, Gopalakrishnan et al. and Matson et al. reported that responders to immunotherapy exhibit higher gut microbial diversity and an enrichment of beneficial taxa [3,27]. Therefore, although direct microbiota data were not captured in our cohort, our findings are consistent with established microbiome-immunotherapy interactions and emphasize the need for future prospective trials that incorporate microbial sequencing, functional metagenomics, and metabolomic analysis to better delineate host–microbiota–drug interactions in RCC.

## 5. Limitations

This study has several limitations that should be acknowledged. First, due to the retrospective design, no direct microbiome profiling (e.g., 16S rRNA sequencing or metagenomic analysis) was available; therefore, any mechanistic link between antibiotic exposure, gut dysbiosis, and impaired immunotherapy efficacy remains speculative and is based on previously published evidence rather than direct biological measurements.

Second, the clinical indications for antibiotic use were not systematically recorded, which may introduce confounding by indication, as patients requiring antibiotics may have had infections or clinical deterioration that independently influenced outcomes.

Third, although no patients in our cohort received systemic corticosteroids or antifungal agents during the peri-immunotherapy period, other potential confounders, including comorbidities, prior lines of systemic therapy, and baseline inflammatory status, were not fully captured and could have influenced survival outcomes.

Another important limitation is the lack of consistent documentation of the clinical indications for antibiotic use. Because this was a retrospective study, it was not possible to reliably determine whether poorer outcomes were attributable to antibiotic exposure itself or to underlying infections that prompted antibiotic administration.

Nevertheless, our real-world cohort offers valuable insights into an underexplored area in RCC and highlights the importance of future prospective trials that include microbiome profiling and antibiotic stewardship strategies.

## 6. Conclusions

Peri-treatment antibiotic exposure was linked to significantly shorter PFS and OS in RCC patients receiving ICIs, with the most pronounced negative effect observed in those with intermediate and poor IMDC risk profiles. These findings reinforce the expanding body of evidence indicating that gut microbiota health is integral to immunotherapy response and highlight the significance of judicious antibiotic use during ICI treatment. This finding has direct implications for antimicrobial stewardship in daily oncology practice.

As one of the few real-world analyses of a Turkish RCC population, this study provides valuable regional data in an area where evidence remains limited. However, its retrospective design means causal relationships cannot be definitively determined. Prospective, multicenter studies incorporating microbiome sequencing and functional profiling are necessary to confirm these findings and better understand microbiota-based predictors of ICI outcomes in RCC.

## Figures and Tables

**Table 1:** Distribution of Antibiotic Classes and Routes of Administration Among Antibiotic-Exposed Patients (*n* = 38).

Antibiotic Class	Number of Patients (*n*)	Percentage (%)	Route of Administration
Fluoroquinolones	12	31.6%	Oral (*n* = 8), IV (*n* = 4)
Beta-lactams	11	28.9%	IV (*n* = 9), Oral (*n* = 2)
Macrolides	6	15.8%	Oral
Carbapenems	3	7.9%	IV
Others/Not specified	6	15.8%	Mixed/Unknown
Total	38	100%	

**Table 2:** Baseline Demographic and Clinical Characteristics of the Study Population.

Characteristic	Total (*n* = 120)	Antibiotic-Exposed (*n* = 38)	Non-Exposed (*n* = 82)
Age (mean ± SD), years	62.4 ± 9.8	61.2 ± 8.5	63.0 ± 10.1
Sex, *n* (%)			
Male	84 (70%)	26 (68%)	58 (71%)
Female	36 (30%)	12 (32%)	24 (29%)
IMDC Risk Group, *n* (%)			
Favorable	22 (18.3%)	6 (15.8%)	16 (19.5%)
Intermediate	68 (56.7%)	21 (55.3%)	47 (57.3%)
Poor	30 (25.0%)	11 (28.9%)	19 (23.2%)
ECOG Performance Status, *n* (%)			
0–1	94 (78.3%)	28 (73.7%)	66 (80.5%)
≥2	26 (21.7%)	10 (26.3%)	16 (19.5%)
Type of ICI Regimen, *n* (%)			
Nivolumab monotherapy	72 (60%)	23 (60.5%)	49 (59.8%)
Combination immunotherapy ± TKI	48 (40%)	15 (39.5%)	33 (40.2%)

Abbreviations: IMDC: International Metastatic RCC Database Consortium; ECOG: Eastern Cooperative Oncology Group; ICI: immune checkpoint inhibitor; TKI: tyrosine kinase inhibitor.

**Table 3:** Comparison of PFS, OS, ORR and DCR between patients exposed to antibiotics and those not exposed, receiving immune checkpoint inhibitors.

Outcome	Antibiotic-Exposed (*n* = 38)	Non-Exposed (*n* = 82)	*p*-Value	Hazard Ratio (95% CI)
Overall Cohort				
Median PFS (months, 95% CI)	5.1 (3.9–6.4)	9.4 (7.8–10.9)	0.004	1.87 (1.21–2.89)
Median OS (months, 95% CI)	14.8 (11.2–18.9)	22.5 (18.0–26.7)	0.03	1.64 (1.04–2.59)
Objective Response Rate (ORR)	18.4%	31.6%	0.09	–
Disease Control Rate (DCR)	47.3%	61.0%	0.12	–
IMDC Intermediate Risk (*n* = 68)				
Median PFS (months)	4.8	8.7	0.006	–
Median OS (months)	13.5	20.6	0.035	–
IMDC Poor Risk (*n* = 30)				
Median PFS (months)	2.9	5.3	0.041	–
Median OS (months)	8.1	13.9	0.049	–

Abbreviations: IMDC subgroup analyses are presented for intermediate- and poor-risk categories. Survival outcomes are illustrated in Figure 1, Figure 2, Figure 3, Figure 4, Figure 5 and Figure 6. HRs and *p*-values are derived from Cox proportional hazards models or log-rank tests.

## Data Availability

The data utilized in this study are available but cannot be shared publicly due to patient confidentiality and privacy considerations.

## References

[1] Routy B., Le Chatelier E., Derosa L., Duong C.P.M., Alou M.T., Daillère R., Fluckiger A., Messaoudene M., Rauber C., Roberti M.P. (2018). Gut microbiome influences efficacy of PD-1-based immunotherapy against epithelial tumors. Science.

[2] Killock D. (2018). Immunotherapy: Gut bacteria modulate responses to PD-1 blockade. Nat. Rev. Clin. Oncol..

[3] Gopalakrishnan V., Spencer C.N., Nezi L., Reuben A., Andrews M.C., Karpinets T.V., Prieto P.A., Vicente D., Hoffman K., Wei S.C. (2018). Gut microbiome modulates response to anti-PD-1 immunotherapy in melanoma patients. Science.

[4] Keen E.C., Crofts T.S., Dantas G. (2018). Checkpoint Checkmate: Microbiota Modulation of Cancer Immunotherapy. Clin. Chem..

[5] Farhadi Rad H., Tahmasebi H., Javani S., Hemati M., Zakerhamidi D., Hosseini M., Alibabaei F., Banihashemian S.Z., Oksenych V., Eslami M. (2024). Microbiota and Cytokine Modulation: Innovations in Enhancing Anticancer Immunity and Personalized Cancer Therapies. Biomedicines.

[6] Derosa L., Hellmann M.D., Spaziano M., Halpenny D., Fidelle M., Rizvi H., Long N., Plodkowski A.J., Arbour K.C., Chaft J.E. (2018). Negative association of antibiotics on clinical activity of immune checkpoint inhibitors in patients with advanced renal cell and non-small-cell lung cancer. Ann. Oncol..

[7] Liao M., Xie Y., Mao Y., Lu Z., Tan A., Wu C., Zhang Z., Chen Y., Li T., Ye Y. (2018). Comparative analyses of fecal microbiota in Chinese isolated Yao population, minority Zhuang and rural Han by 16sRNA sequencing. Sci. Rep..

[8] Gupta V.K., Paul S., Dutta C. (2017). Geography, ethnicity or subsistence-specific variations in human microbiome composition and diversity. Front. Microbiol..

[9] Okalin S.S., Arslan N., Demiray Gürbüz E., Arayıcı M., Kırca N.D., Ozel Demiralp D., Dereli-Akdeniz D., Akan P., Ozkutuk A.A. (2025). The association between gut microbiota composition and cardiometabolic parameters in healthy adults. BMC Microbiol..

[10] York A. (2018). Microbiome: Gut microbiota sways response to cancer immunotherapy. Nat. Rev. Microbiol..

[11] Vetizou M., Trinchieri G. (2018). Anti-PD1 in the wonder-gut-land. Cell Res..

[12] Pinato D.J., Howlett S., Ottaviani D., Urus H., Patel A., Mineo T., Brock C., Power D., Hatcher O., Falconer A. (2019). Association of Prior Antibiotic Treatment with Survival and Response to Immune Checkpoint Inhibitor Therapy in Patients with Cancer. JAMA Oncol..

[13] Yang Z., Wei S., Liu L. (2020). Antibiotic Treatment and Immune Checkpoint Inhibitor Therapy in Patients with Cancer. JAMA Oncol..

[14] Iglesias-Santamaría A. (2020). Impact of antibiotic use and other concomitant medications on the efficacy of immune checkpoint inhibitors in patients with advanced cancer. Clin. Transl. Oncol..

[15] Belkaid Y., Hand T.W. (2014). Role of the microbiota in immunity and inflammation. Cell.

[16] Thaiss C.A., Zmora N., Levy M., Elinav E. (2016). The microbiome and innate immunity. Nature.

[17] Motzer R.J., Escudier B., George S., Hammers H.J., Srinivas S., Tykodi S.S., Sosman J.A., Plimack E.R., Procopio G., McDermott D.F. (2020). Nivolumab versus everolimus in patients with advanced renal cell carcinoma: Updated results with long-term follow-up of the randomized, open-label, phase 3 CheckMate 025 trial. Cancer.

[18] Yang M., Wang Y., Yuan M., Tao M., Kong C., Li H., Tong J., Zhu H., Yan X. (2020). Antibiotic administration shortly before or after immunotherapy initiation is correlated with poor prognosis in solid cancer patients: An up-to-date systematic review and meta-analysis. Int. Immunopharmacol..

[19] Nie F., Guo J., Pan J., Guo Z., Wang C., Yan J., Ma W. (2025). Effects of antibiotics on the anti-tumor efficacy of immune checkpoint inhibitor therapy. Clin. Transl. Oncol..

[20] Alotaibi F.M., Albalawi I.A.S., Anis A.M., Alotaibi H., Khashwayn S., Alshammari K., Al-Tawfiq J.A. (2024). The impact of antibiotic use in gastrointestinal tumors treated with immune checkpoint inhibitors: Systematic review and meta-analysis. Front. Med..

[21] Huang C., Li M., Liu B., Zhu H., Dai Q., Fan X., Mehta K., Huang C., Neupane P., Wang F. (2021). Relating Gut Microbiome and Its Modulating Factors to Immunotherapy in Solid Tumors: A Systematic Review. Front. Oncol..

[22] Piao X.M., Byun Y.J., Zheng C.M., Song S.J., Kang H.W., Kim W.T., Yun S.J. (2023). A New Treatment Landscape for RCC: Association of the Human Microbiome with Improved Outcomes in RCC. Cancers.

[23] Yu Y., Zheng P., Gao L., Li H., Tao P., Wang D., Ding F., Shi Q., Chen H. (2021). Effects of Antibiotic Use on Outcomes in Cancer Patients Treated Using Immune Checkpoint Inhibitors: A Systematic Review and Meta-Analysis. J. Immunother..

[24] Huang L., Li Y., Zhang C., Jiang A., Zhu L., Mou W., Li K., Zhang J., Cui C., Cui X. (2025). Microbiome meets immunotherapy: Unlocking the hidden predictors of immune checkpoint inhibitors. npj Biofilms Microbiomes.

[25] Derosa L., Iebba V., Albiges L., Fidelle M., Bonvalet M., Colomba E., Zitvogel L., Escudier B., Routy B. (2018). Gut microbiome composition to predict resistance in renal cell carcinoma (RCC) patients on nivolumab. J. Clin. Oncol..

[26] Pinato D.J., Gramenitskaya D., Altmann D.M., Boyton R.J., Mullish B.H., Marchesi J.R., Bower M. (2019). Antibiotic therapy and outcome from immune-checkpoint inhibitors. J. Immunother. Cancer.

[27] Matson V., Fessler J., Bao R., Chongsuwat T., Zha Y., Alegre M.L., Luke J.J., Gajewski T.F. (2018). The commensal microbiome is associated with anti-PD-1 efficacy in metastatic melanoma patients. Science.

